# *AlphaFold* and the future of structural biology^1^

**DOI:** 10.1107/S2052252523004943

**Published:** 2023-06-26

**Authors:** Randy J. Read, Edward N. Baker, Charles S. Bond, Elspeth F. Garman, Mark J. van Raaij

**Affiliations:** a Cambridge Institute for Medical Research, University of Cambridge, The Keith Peters Building, Hills Road, Cambridge CB2 0XY, United Kingdom; b School of Biological Sciences, University of Auckland, Auckland, New Zealand; c School of Molecular Sciences, University of Western Australia, 35 Stirling Highway, Crawley, WA 6009, Australia; d Department of Biochemistry, University of Oxford, Dorothy Crowfoot Hodgkin Building, South Parks Road, Oxford OX1 3QU, United Kingdom; e Departamento de Estructura de Macromoleculas, Centro Nacional de Biotecnologia, Consejo Superior de Investigaciones Cientificas, 28049 Madrid, Spain

**Keywords:** *AlphaFold*, protein structure prediction, crystallography, cryo-EM

## Abstract

This editorial acknowledges the transformative impact of new machine-learning methods, such as the use of *AlphaFold*, but also makes the case for the continuing need for experimental structural biology.

The landscape of structural biology changed dramatically in 2020 with the emergence of the second version of the *AlphaFold* protein structure-prediction program (Jumper *et al.*, 2021 ▸). Since then, structural biologists, and the many other scientists whose research is informed by knowledge of macromolecular structure, have scrambled to adapt. Much of the hype in both the scientific literature and popular media has indeed been justified: the current version of *AlphaFold* is astonishingly powerful, and it would be foolish not to make full use of it. Unfortunately, recent anecdotal reports suggest that some scientists reviewing grants and papers believe that *AlphaFold* is so powerful that it has made experimental structural biology redundant. There is clearly still a need for education about the strengths and weaknesses of current machine-learning methods for protein structure prediction, and the essential role that remains for experiment.

The advance in *AlphaFold* was unveiled at CASP14, the 14th edition of the Critical Assessment of protein Structure Prediction. For the first time in the history of CASP, the best models of protein targets were judged as being competitive with experimental structures in their backbone accuracy, almost independent of the difficulty of the modelling problem (Kryshtafovych *et al.*, 2021 ▸). Since then, other powerful machine-learning algorithms for structure prediction have emerged, including the first (Baek *et al.*, 2021 ▸) and second (Baek *et al.*, 2023 ▸) versions of *RoseTTAFold*, *ESMfold* (Lin *et al.*, 2023 ▸) and *OpenFold* (Ahdritz *et al.*, 2022 ▸), an open-source reimplementation of *AlphaFold* including training code.

Within half a year of CASP14, the *AlphaFold* algorithm was made freely available through Google Colab notebooks, including a community-developed version (Mirdita *et al.*, 2022 ▸). Pre-calculated structures of a large collection of proteins from important genomes were made available through the AlphaFold Database at the European Bioinformatics Institute (Tunyasuvunakool *et al.*, 2021 ▸), which has since been expanded to include about 200 million proteins representing most of the UniProt database (Varadi *et al.*, 2022 ▸).

Structural biologists quickly embraced the advantages that these models bring (Perrakis & Sixma, 2021 ▸), including insights into improved construct design, the generation of compelling hypotheses about interacting proteins or domains, and the acceleration of structure solution by molecular replacement (Millán *et al.*, 2021 ▸) for crystallography or docking for electron cryo-microscopy (cryo-EM). A particularly notable example was integrative modelling of the nuclear pore complex by combining *AlphaFold* (Jumper *et al.*, 2021 ▸) and *RoseTTAFold* (Baek *et al.*, 2021 ▸) models with cryo-electron tomography and complementary data (Mosalaganti *et al.*, 2022 ▸).

Most structural biologists are aware that the models are not actually as accurate as experimental structures. The backbone accuracy measured in CASP does not ensure the accuracy of all coordinates including side chains. An objective evaluation of how well different models explain experimental diffraction data showed that experimental structures from alternative crystal forms (in spite of different crystal-packing interactions) are generally better than *AlphaFold* models (Terwilliger *et al.*, 2023 ▸). It has also been reported that *AlphaFold* models perform less well than experimental structures as targets for the computational docking algorithms used in drug design (Karelina *et al.*, 2023 ▸).

It is also important to remember that multi-domain targets in CASP are subdivided into ‘evaluation units’ when predictors fail to predict their relative orientations (Kinch *et al.*, 2021 ▸). Many *AlphaFold* models, including those submitted to CASP14, indeed have large errors in the relative orientations of domains.

The authors of *AlphaFold* acknowledge its limitations, and one of the great strengths of *AlphaFold* is its ability to assess its own accuracy: the predicted aligned error (PAE) matrix presents clear warnings when relative domain orientations are uncertain, and the predicted value of the local distance difference test or LDDT (Mariani *et al.*, 2013 ▸) statistic (pLDDT) correlates rather well with the actual local model accuracy (Jumper *et al.*, 2021 ▸).

The most serious limitations of *AlphaFold* and other machine-learning algorithms for structure prediction arise from the fact that they are based on learning patterns and know almost nothing about physics and chemistry. They can generate a single structure that is most consistent with the patterns they know about, but not a collection of alternative conformations that are influenced in their relative stability by factors such as pH, temperature or the binding of ions, other ligands or other proteins. For the foreseeable future, we will certainly need experiments to assess these effects. In addition, variants with amino-acid substitutions will satisfy the same patterns, even though such substitutions may destabilize the protein or change its conformation. Nonetheless, other algorithms exist that can use the *AlphaFold* model to predict the effects of variants on stability as accurately as with an experimental structure (Akdel *et al.*, 2022 ▸).

One of the most powerful aspects of experimental structural biology is the discovery of the unexpected, including the discovery of obligate cofactors and specific metal ions in structures, as well as structurally important post-translational modifications, including various kinds of spontaneous chemical cross-linking such as is observed in fluorescent proteins. Crystallography and cryo-EM will also give an answer to the question of stoichiometry of a homomer (or heteromer), while *AlphaFold*, despite valuable developments in multimer prediction (Evans *et al.*, 2022 ▸), still requires hypothetical stoichiometries to be tested.

Papers describing macromolecular structure-prediction algorithms and their use are welcome in the relevant IUCr journals: *Structural Biology* (*Acta Crystallographica Section D*), *Structural Biology Communications* (*Acta Crystallo­graphica Section F*) and *IUCrJ*. For new structures, authors should bear in mind that the use of predicted structures as molecular-replacement models has already become standard practice, and papers describing the resulting structures must make new contributions to our biological understanding.

The field of structural biology and the nature of its challenges has, for the second time this century, undergone a major change. The resolution revolution in cryo-EM has allowed previously intractable systems to be studied at resolutions permitting ‘*de novo*’ model building, but it has not made X-ray crystallography or NMR spectroscopy redundant (Kühlbrandt, 2014 ▸). In the same way, the major leap in structure prediction that *AlphaFold* has spawned does not mean that experimental techniques have suddenly become meaningless. As other commentators have said (Perrakis & Sixma, 2021 ▸; Kleywegt & Velankar, 2022 ▸), this change is accelerating progress and bringing us more power to address deeper questions, and we should embrace the new possibilities.
